# Definition and developmental goals for Nordic emergency medicine

**DOI:** 10.1186/s13049-025-01319-0

**Published:** 2025-01-14

**Authors:** Hjalti Már Björnsson, Ari Palomäki, Christian Baaner Skjærbæk, Frederik Pors Klinting, Frida Meyer, Jørn Einar Rasmussen, Lars Petter Bjørnsen

**Affiliations:** 1 Department of Emergency Medicine, Landspitali–The National University Hospital of Iceland, Reykjavík, Iceland; 2 University of Iceland, Reykjavík, Iceland; 3 Professor of Emergency Medicine, Faculty of Medicine and Health Technology, Tampere University, Tampere, Finland; 4 Emergency Department, Kanta-Häme Central Hospital, Kanta-Häme Wellbeing District, Hämeenlinna, Finland; 5 Regionshospitalet Randers, Randers, Denmark; 6 Sygehus Lillebælt, Kolding, Denmark; 7 Department of Emergency Medicine, Linköping University Hospital, Linköping, Sweden; 8 Emergency and Preparedness Department, Skien Hospital Trust, Skien, Norway; 9 Department of Emergency Medicine, St. Olav’s Hospital, Trondheim, Norway; 10 Dept of Department of Circulation and Medical Imaging, Faculty of Medicine, Norwegian University of Science and Technology (NTNU), Trondheim, Norway; 11 Department of Biomedical and Clinical Sciences, Linköping University, Linköping, Sweden

## Abstract

Although Emergency Medicine is now globally an established specialty, the Nordic countries have been relatively slow to implement it into their health care systems. To facilitate the development of EM in the Nordic area, a working group was formed with representation from all national EM societies; DASEM (Danish Society for Emergency Medicine), FiSEM (Finnish Society of Emergency Medicine), ISEM (Icelandic Society for Emergency Medicine), NCEM (Norwegian College of Emergency Medicine), and SWESEM (Swedish Society for Emergency Medicine). This group was tasked with creating a Nordic EM manifesto—to create a definition and developmental goals for Nordic Emergency Medicine. The commentary provides an overview of the current status and challenges facing EM in the Nordic countries.

Emergency medicine (EM) is a specialty that focuses on the initial evaluation and stabilization of acute patients in the emergency department (ED) or in the prehospital setting. It is based on having fully trained emergency physicians available 24/7 in the ED who have the required knowledge and skills to evaluate and treat all patient populations and acute presentations and to perform any emergency procedures that are technically feasible to perform in the ED [1]. This contrasts significantly with the earlier model of having trainees from various departments in each hospital run down to the ED to manage acute patients who are presumed to have a problem related to their respective specialty.

Globally, EM is a fully developed independent medical specialty that has been completely integrated into the healthcare system of most countries for years or decades. Although this has progressed at a variable pace in different countries over the last 50 years, there are no countries or hospitals that have fully established EM as a medical specialty and then later decided to revert back to the older ED model. Like in all other departments of a hospital, having the ED staff with specialized doctors is currently considered the standard of providing EM care [1].

Nordic countries have generally been slow to implement EM in their health care systems. Iceland was the first to officially recognize EM as an independent specialty in 1992, followed by Finland in 2013, Sweden in 2015, and finally Denmark and Norway in 2017 and 2019, respectively [2–6]. Although all Nordic counties currently recognize EM as an independent specialty, training a sufficient number of physicians to staff EDs in all countries and tasking them with providing acute care according to international EM standards has, to date, been achieved only partially in the area [7].

This delay may be partially caused by a lack of collaboration or political will to substantiate the organisational changes required to fully implement EM. Despite the Nordic countries being culturally and politically similar and having comparable healthcare systems, collaboration in establishing EMs has been limited. Each Nordic country has a national EM society that has developed its own separate curriculum and pathway for EM training. It has been relatively uncommon for EM trainees to perform an elective period of training in another Nordic country, and few common Nordic training courses in EM have been held. A common Nordic conference on EM has not been arranged, and few individuals have obtained EM training abroad in a health care system with EM care fully established according to the EM model.

The lack of collaboration is also likely to have contributed to the current state of variation in EM physicians’ scope of practice, both between different Nordic countries and different hospitals within each country.

One specific problem is the lack of common terminology. Unfortunately, in all Nordic languages except for Icelandic, the term “emergency physician” (akuttlege/akutläkare/akutlæge/akuuttilääkäri) has quite often been used for any doctor working in the ED, regardless of what specialty training that a particular doctor has. For the rest of the world, this is considered absurd as emergency physicians to be called cardiologists just because they happen to work in the cardiology department. Importantly, Nordic countries should adapt to the global standard of using the term “emergency physician” only for doctors with full specialty training in EM.

Another significant problem that has been encountered with establishing EM in Nordic countries is that EPs have often been tasked with providing inpatient care to manage admitted patients in short stay units for even up to 2–3 days. This is not within the scope of practice for EM and significantly hinders the focus of EM – to immediately evaluate and stabilize acute patients and then refer them to further care by other specialties if needed. EMs should generally manage patients for up to 4‒6 h, except for highly selected patients, who might benefit from ED observation and stabilization for up to 24 h.

As a result of the outlined discussion above, the Nordic EM societies formed a working group in the fall of 2023 to create a concise common Nordic definition for EM. The goal was to create common definitions and developmental goals for EM in the area on the basis of earlier similar publications and definitions from co-operation of American organisations in the USA as well as European Union of Medical Specialists, Section for Emergency Medicine, and European Society for Emergency Medicine in Europe[1, 8, 9]. Hopefully, this will facilitate the development and standardization of EM in our area.

The definition has been discussed and formally approved by the leadership of all Nordic EM societies.

1. Emergency physicians:
  - An emergency physician is a physician who has completed an accredited specialty training in emergency medicine and is certified as a specialist in emergency medicine by national or local authorities.
  - Should be able to perform the initial evaluation and treatment of all patients presenting to the emergency department or in the pre-hospital setting.
  - Should have training to perform any acute procedures that are technically feasible to perform in the emergency department or on scene and required to stabilize unstable patients in accordance with European core curriculum for emergency medicine and European training requirements for the specialty of emergency medicine or equivalent ones [8, 9]
  - Physicians with other specialty training working in the emergency department should not be referred to as emergency physicians but as specialists or trainees in whatever field they are certified in.
  - Physicians in specialty training in emergency medicine should be referred to as emergency medicine trainees, not emergency physicians.
2. An emergency department should:
  - Be an independent department within the hospital, led by an emergency physician.
  - Always be staffed with fully trained emergency physicians supervising EM trainees.
3. In all hospitals with emergency departments, emergency physicians should:
  - Be in charge of the medical care provided in the emergency department, including defining the roles of other physicians that may be called on to participate in patient management in the ED.
  - Collaborate with nurses, physiotherapists, pharmacists and other health care professionals working in the ED, and consulting physicians from other specialties who may be called on to provide specific management of selected patients in the ED.
  - Fully evaluate, treat and discharge selected patients in the ED.
  - Refer patients in need of further evaluation or management by other specialties to the suitable in- or outpatient services within the appropriate timeframe.
  - Generally, manage patients only for the initial evaluation and stabilization, and observation of selected emergency patients for up to 24 h.

## Data Availability

No datasets were generated or analysed during the current study.
